# Emerging liquid biopsy tools to analyse cancer biomarkers: electrochemical sensors and extracellular vesicle analysis

**DOI:** 10.1042/BST20253109

**Published:** 2026-02-18

**Authors:** Anitha Devadoss, Michael R. Furness, Nathan J.W. Wu, Olga Oikonomidou, Maïwenn Kersaudy-Kerhoas, Nick R. Leslie

**Affiliations:** 1 Institute of Biological Chemistry, Biophysics and Bioengineering, School of Engineering and Physical Sciences, Heriot-Watt University, Edinburgh EH14 4AS, U.K.; 2 Cancer Research UK Edinburgh Centre, Institute of Genetics and Cancer, University of Edinburgh, Edinburgh, EH4 2XR, U.K.

**Keywords:** cancer biomarkers, electrochemical biosensors, extracellular vesicles, point-of-care diagnosis, proteins

## Abstract

Better biomarker analysis technologies can provide improvements in the detection, characterisation and monitoring of cancer and less invasive sampling of blood and other body fluids can improve acceptability and affordability. Here, we discuss these technologies with a specific focus on recent advances in electrochemical sensors, specifically for the analysis of extracellular vesicles (EVs). Widely used biomarker tests with relatively high sensitivity (e.g. ELISAs) are limited by their cost, storage requirements and shelf-life, and ease of use away from centralised facilities. Moreover, their limits of detection (most commonly in the nanomolar to picomolar, with new technologies pushing into the femtomolar range) remain challenged by low abundance biomarkers. Here, we discuss how electrochemical sensor platforms, although often requiring more effort to adapt for new analytes, can provide high sensitivity and direct quantitation at low cost. These platforms are also often simpler to use away from testing facilities. Additionally, we explore how EVs, by protecting nucleic acid and protein cargos from degradation, may facilitate the collective enrichment from blood samples of multiple tumour-derived biomarkers. Continued progress in analysis technologies, alongside a deeper understanding of biomarker biology and clinical value, holds the potential to improve outcomes for the increasing numbers of individuals diagnosed with cancer.

## Introduction

Driving earlier cancer detection and reducing diagnosis times have clear benefits to successful treatment and patient outcomes. Therefore, developing sensitive diagnostic and monitoring systems for the rapid detection of cancer biomarkers has become a strategic priority of healthcare delivery systems. By delivering and mixing material from tissues throughout the body, blood provides a potential source of informative biomarkers released by cancer cells and tumour stromal cells (in addition to small numbers of these cells themselves) without requiring invasive tissue biopsies. Sampling blood has the advantage of capturing materials released from cancerous tissues regardless of their location within the body. However, while it does not reveal where biomarkers have entered the bloodstream, blood-based analysis provides a minimally invasive route for rapid detection of cancer biomarkers.

Human blood contains several classes of biomarker which can provide insight into potential cancers, including circulating tumour cells (CTCs), extracellular vesicles (EVs), DNA, RNA, proteins and metabolites, each with technologies to analyse them. Several excellent reviews have focused on CTCs, circulating tumour DNA and technologies to analyse these biomarkers, which also address the limitations of utilising these biomarkers for successful cancer diagnosis [1–3]. Here, we highlight recent developments, opportunities and challenges in blood biomarker analysis technology which have the potential to expand the numbers already applied in the clinic, with some focus on electrochemical sensors. We also discuss the implications of the encapsulation of many biomarkers within EVs released by tumour cells and the technologies being developed to exploit them.

And it is important to note throughout, that biomarker tests are evaluated by their clinical value, defined by their sensitivity and specificity (meaning their ability to avoid false negatives and false positives, respectively) and the value of this information in making clinical decisions. Test sensitivity and specificity are influenced by both the strength of the association of the measured biomarker with a specific patient characteristic or pathology in addition to the technical performance of the test. In this review, after an initial introduction to blood-borne cancer biomarkers, we will discuss recent advances in the methods used to detect and quantify them with a focus on electrochemical sensors. We examine the technical strengths and limitations of these technologies, considering factors such as sensitivity, quantitative capability, signal linearity, reproducibility, speed, ease of use, cost and patient acceptability. But, it should not be forgotten that although we address technology, the performance of any biomarker detection method must fit its clinical context.

## Biomarkers within blood

**Figure 1:**
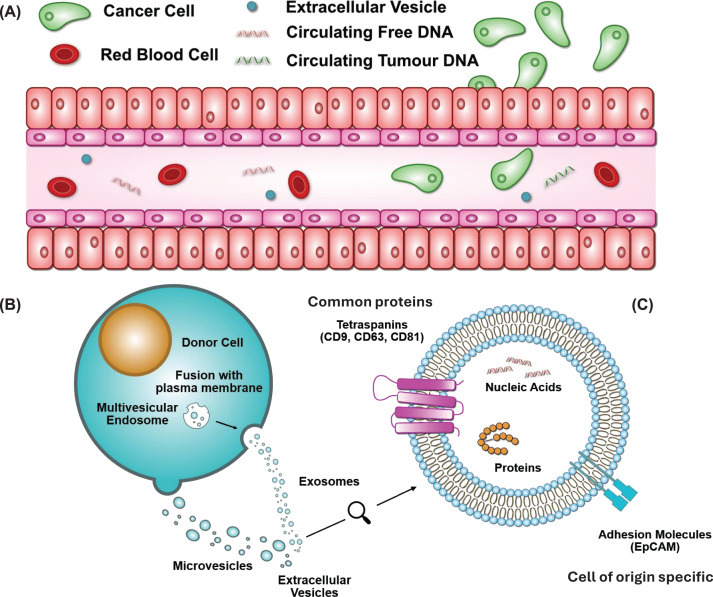
Biomarkers in blood (**A**) A simplified illustration of a blood vessel is shown, identifying cells (also known as CTCs) and DNA. EVs are also represented, which as described in the text are released by potentially all cell types, including cells from diverse organ systems, cancer cells, endothelial cells etc. (**B**) A model cells is represented, showing the release of EVs through both exosomal and microvesicular routes. (**C**) A model EV is shown illustrating examples of shared surface proteins (e.g. Tetraspanins) and proteins which are more specific identifiers for certain cells of origin (e.g. EpCAM on EVs produced by epithelial cells). Internal protein and nucleic acid cargos are also represented.

The circulatory system provides a route to distribute and access many informative bioanalytes originating from locations throughout the body, providing potential insight into healthy organ function, chronic disease, infection etc. (Figure 1). Additionally, in cancer patients this provides opportunities to identify multiple classes of analyte released from both primary tumours and metastases.

### Circulating tumour cells

Cancerous and stromal cells disseminating from a primary tumour into blood vessels and lymphatics has been intensively studied both to understand more deeply the process of metastasis but also as valuable biomarkers. Opportunities to identify and isolate CTCs are provided in many cases by surface proteins and glycoproteins which are absent from blood cells as well as their frequently larger size and lower deformability relative to blood cells. CTC numbers are variable and unpredictable but correlate with disease burden. Late-stage patients often display 5–10 CTCs per ml of blood, with even higher numbers often observed near end of life, yet early-stage cancers often having no detectable CTCs in a sample [4]. Metastatic processes are diverse, but evidence has emerged that CTC time in the circulation is often short (<1 h) and that the greatest risk is posed by cancer cell clusters, particularly heterotypic clusters which contain both cancer and stromal cells [5].

### Protein biomarkers

Recent large scale proteomic studies identify 3000–5000 different proteins in human plasma [6,7], and it has long been known that proteins in plasma represent an extremely rich biomarker resource. Accordingly, several plasma protein biomarkers are in widespread clinical use to detect and characterise cancers. For example, PSA (prostate-specific antigen) and CA125 (Carbohydrate or Cancer Antigen 125) are indicators of prostate and ovarian cancer respectively [8–10]. However, these proteins do not correlate perfectly with disease burden, limiting their clinical reliability. Accordingly, current research includes strategies to improve these situations. Firstly, current efforts to improve the sensitivity of detection of plasma proteins should expand the number of plasma proteins which can yield data of diagnostic value, including proteins which are potentially more disease-selective and more confidently support decision making during treatment [11]. Secondly, the multiomic combination of plasma protein analysis with that of other biomarker classes should also support more personalised and more effective treatment.

### Nucleic acid: circulating cell-free DNA and RNA (cfDNA and cfRNA) including circulating tumour DNA (ctDNA) and micro-RNA (miRNA)

Each millilitre of human blood typically contains several billion red blood cells, several million white blood cells and a few thousand copies of the genome in a fragmented cell-free state, commonly with a modal peak at 166 bp in length. Various physiological insults and pathological conditions can increase the abundance of cfDNA and in particular cancer patients frequently show elevated levels of cfDNA, some of which is derived directly from cancer cells. This subset, known as circulating tumour DNA (ctDNA) can reveal the mutations, methylation patterns and genomic copy number changes found in those cancer cells. The clinical exploitation of ctDNA biomarkers is well-established with many products and analytical methods in frequent use. However, challenges remain, particularly in managing costs and in sensitivity in early-stage cancers without prior sampling of cancer tissue. These issues have been comprehensively reviewed by others [1,3].

Similarly, diverse classes of cellular RNAs (mRNAs, miRNAs, cRNAs, rRNA etc.) are also present in blood. The total yield of cfRNA is often comparable to the yield of cfDNA, in the 5–10 ng/ml range. Notably, a significant proportion of cfDNA and particularly cfRNA is encapsulated in EVs (see below) protecting it from degradation by nucleases [12]. Due to this encapsulation and their stability, miRNAs have emerged as promising biomarkers. Advances in data analysis methods, many driven by artificial intelligence, are helping to overcome initial challenges in the identification of cancer-specific miRNA signatures. To date, over 64 miRNAs have been identified as potential markers for hard to diagnose cancers, with reported sensitivities ranging from 72.5% to 100% and specificities from 73% to 100% [13].

### Extracellular vesicles

EVs are small volumes of cellular material encased in a single lipid bilayer membrane, and are found abundantly throughout the body, secreted by potentially all cell types, including healthy and cancerous cells [14]. Their cargos include proteins, DNA and RNA and they appear to fulfil roles in intercellular communication: for example, mRNA and miRNA delivered by EVs can be expressed in, and/or influence the behaviour of, cells that take up such EVs [14,15].

EVs are often classified by their size as exosomes (40–150 nm) or microvesicles (100–1000 nm) and also based on their source, with some being identified as apoptotic bodies. The understanding of the diversity of EVs and their production and cargo selection is developing rapidly. However, it is clear that populations of exosomes can be produced by internal membrane budding into late endosomes and released through fusion of the resultant multivesicular body with the cell membrane (see Figure 1). Microvesicles in contrast can be formed by direct budding from the cell membrane and apoptotic bodies are produced during cell death through membrane blebbing and cell fragmentation.

It is thought EVs share the same EV-membrane protein repertoire, e.g., CD9, CD63 and CD81 being present on the vast majority of EVs [16,17]. Additionally, tumour-derived EVs exhibit distinctive membrane proteins that are specific to their cellular origin e.g. cancer biomarkers human epidermal growth factor receptor-2 (HER2), epidermal growth factor receptor (EGFR) and epithelial cell adhesion molecule (EpCAM) [18]. Tumour-derived EVs have recently emerged as an important class of circulating biomarkers for cancer diagnosis due to their existence in numerous biofluids. For example, saliva- and serum-derived EVs can be employed for the diagnosis of breast cancer (BrCa) [19]. Moreover, a single tumour cell can release more than 10^4^ EVs per day, making tumour derived EVs more abundant (10^7^–10^9^ EVs/mL of blood) than other circulating biomarkers such as CTCs (typically <10 CTCs/ml blood) [20,21]. Thus, widespread tumour-derived EV concentrations naturally occurring in blood as well as their unique surface protein signatures make them promising biomarkers.

Furthermore, recent preclinical reports demonstrate that profiling of EV-bound surface proteins also offers key information on critical processes for cancer progression and metastasis. For instance, Wang et al. showed that increased levels of transient receptor potential channel 5 (TRPC5) protein in plasma EVs can predict a low response to chemotherapy prior to tumour progression, determined by imaging examination [22]. Ciravolo et al. demonstrated that HER2 positive EVs secreted from HER2 overexpressing BrCa cell lines could sequester the therapeutic antibody trastuzumab leading to drug resistance, but did not show binding to, or effect treatment with, the small molecule HER2 inhibitor lapatinib [23]. Thus, developing detection technologies for the precise detection of EVs has high clinical significance in both cancer diagnostics as well as therapeutics.

### Other circulating biomarkers: oncometabolites, platelets, and circulating stromal cells

Several other components of blood have seen investigation as potential biomarkers. The reprogramming of cellular metabolism that is commonly a central part of cellular transformation can cause detectable changes in the concentration of specific ‘oncometabolites’ in plasma such as 2-hydroxyglutarate in cancers carrying driver mutations in isocitrate dehydrogenase enzymes [24]. Furthermore, additional cell types, including circulating tumour-derived stromal cells [25] and platelets have seen investigation, with particular interest in platelets driven by their uptake of circulating cfDNA [26] as well as proteins and nucleic acid specifically from cancer cells, leading to the label ‘tumour educated platelets’ [27].

## Technologies to isolate circulating biomarkers

Many analytical methods incorporate enrichment or isolation of analytes prior to detection and quantitation. In some cases, this is a necessity: for example, a nucleic acid purification step is required prior to almost all PCR and DNA sequencing methods and purification of total cell-free nucleic acid from blood is common. In other methods, such as capture sequencing or sandwich ELISA, selective target binding is an integral part of the method. An example of this that has seen considerable recent attention paid to it is the enrichment of EVs, and potentially EVs from specific tissues or with specific characteristics, from blood and other body fluids [28,29]. In cancer biomarker analysis, this has the advantages of increasing some biomarker detection sensitivities, as well as providing insight into a collection of multiple characteristics through combining analysis of multiple protein and/or nucleic acid EV cargos.

### Isolating circulating tumour cells

Methods to enrich, isolate and quantify CTCs from blood are well-developed, with several diverse technologies available commercially [30]. Notably, in some settings cells are isolated for further analysis, whereas in others, measuring the number of CTCs itself is used as an indicator of disease burden and response to therapy. Approaches include affinity purification methods based on the expression of cell-surface markers which are absent from blood cells, such as EpCAM and Cytokeratin (e.g. CellSearch), as well as devices sited intravenously to capture cells directly from circulating blood (e.g. CellCollector). These marker-based approaches have proved powerful in some settings, but not all cancers express markers such as EpCAM and these methods also have been shown to isolate marker-positive epithelial cells from individuals with benign disease [31,32]. Other methods of CTC isolation include those based on their generally larger cell size relative to blood cells, their lower deformability and their lower density. These systems include the Parsortix system relying on size and deformability, which has been shown to perform relatively well in comparative testing [33].

Evaluation of CTC numbers has proved to be a valuable prognostic indicator in several cancer types and to change with disease progression and successful treatment [34,35] and single-cell ‘omics technologies can be applied well to CTC populations. Furthermore, many research groups have successfully developed cell cultures from individual CTCs, not only to investigate cancer cell biology more deeply but also to test potential therapies *in vitro* over a period of weeks with the potential to inform treatment of the originating patient. However, such studies are limited by low CTC numbers and the low frequency with which these cells initiate viable cultures, which is usually <15%. The low numbers of CTCs present in blood samples from early-stage cancer patients can also raise questions about how data (e.g. from <5 cells) reflects genomic and phenotypic diversity within the patient’s cancer.

### Isolating extracellular vesicles

A wide range of technologies have been developed to isolate and enrich EVs based on their size, charge, and affinity [36–40]. Ultracentrifugation is the most used (gold standard) large volume technology for the isolation of EVs in research, which uses differential centrifugal forces to remove debris and sediment EVs. However, the need for bulky and costly instrumentation, laborious processing, and the need for the large sample volumes limits its practical application in clinical settings. Alternate separation techniques including gradient ultracentrifugation, polymer precipitation, and field flow fractionation are also being employed for EV isolation which often results in high purity EV isolates, but low yield and low throughput need to be addressed. Accordingly, more easily applicable microfluidic technologies incorporating vesicle capture by antibodies and aptamers recognising EV surface proteins have also been successfully employed for EV isolation. Sieving, trapping in porous substrates, hydrodynamic isolation, and dielectrophoresis have also been explored [41–44].

The high level of heterogeneity of EVs in size, origin, and molecular constituents makes the isolation and enrichment of EVs, or sub-populations of EVs, from clinical samples of great benefit for biomedical investigation, providing information not available from total plasma or serum. Furthermore, enrichment of EVs based on one marker, such as a surface protein, can be combined with analysis of additional EV-associated proteins, nucleic acid sequences etc. Methods including affinity capture and the analytical growth of gold nanoshells on EVs allow the discrimination of proposed cancer biomarker proteins on EVs from these proteins free in serum [37,39]. And a further example of this multiparametric analysis is the demonstration of lab-on-a-chip immunoaffinity capture of EVs from cancer patient blood samples combined with analysis of EV-associated matrix metalloprotease (MMP14) activity which was able to predict the invasiveness of individual cancers [36].

### Technologies to analyse circulating biomarkers

### PCR and sequencing of nucleic acid (cfDNA and cfRNA)

The genomes of cancer cells carry changes that can be detected and are potentially clinically informative including point mutations, insertions, deletions and chromosomal translocations as well as other changes such as in DNA methylation patterns. Therefore, this DNA when present in the circulation, mostly in small fragments (160–170 bp), reflects these informative changes in sequence, copy number and methylation. Critical factors for the detection and analysis of circulating tumour DNA include firstly that it exists in a background dominated by wild-type cfDNA derived from the body’s non-cancerous cells and in early-stage cancer patients, this fraction of mutated informative DNA (often termed the variant allele fraction or VAF) is very low, usually <1%. And secondly, that routine purification methods for nucleic acid from plasma include detergents which disrupt EVs so that purified cfDNA and cfRNA comprises both EV-associated, protein-associated and any more genuinely ‘free’ nucleic acid.

The most commonly applied methods for the analysis of circulating tumour DNA are forms of sequencing, often targeted, and of PCR (polymerase chain reaction), often mutation-specific PCR. Importantly, some methods such as allele-specific digital PCR, are extremely sensitive but detect individual mutations, so are most useful in tumour-informed circumstances in which mutations have already been identified (e.g. monitoring for relapse by detection of known mutations), or in which specific mutations are known to be very common (e.g. BRAF V600E mutations in melanoma). Very recently tumour-informed whole genome cfDNA sequencing has been reported to detect ctDNA with a sensitivity approaching 1 ppm (0.0001% VAF) with excellent specificity [45].

dPCR of ctDNA is also performed to inform individual therapeutic decisions based on the identification of specific mutations (e.g. KRAS G12C testing to select patients for treatment with the mutation-specific inhibitor Sotorasib). In contrast, other sequencing methods applied to cfDNA, such as shallow genome sequencing to identify copy number variation, or targeted sequencing of frequently mutated sites are tumour agnostic, not requiring prior knowledge of the sample. Furthermore, additional characteristics of cfDNA can be exploited, including DNA fragment size, end motifs, and methylation which have distinct disease-associated patterns [46,47]. And enrichment strategies using for example targeted nucleases can also increase the sensitivity of ctDNA detection [48–50]. The analysis of ctDNA and its clinical utility have been reviewed well by others [1–3].

Changes in circulating cfRNA are also observed in cancers, driven both directly and indirectly by developing cancer cells. Similar analytical methods can be used to analyse cfRNA as for cfDNA, specifically RNA sequencing and more focused quantitative PCR. These approaches almost all use reverse transcription to create DNA copies of RNA samples prior to analysis, although direct RNA sequencing is becoming more common through the use of nanopore sequencing [51,52]. Current research efforts are providing both a deeper insight into the broad diversity of cfRNA populations and identifying specific RNA biomarkers associated with individual cancer types [53–55]. However, several challenges influence the consistent analysis of cfRNA. These include the different stabilities of different classes of RNA, differential protection within vesicles, in addition to the variability in the removal of platelets during sample processing, which both contain abundant RNA and can release RNA-rich vesicles after blood-draw [56,57].

### Why do we need better technologies to analyse non-nucleic acid circulating biomarkers?

In many cases, the performance of technologies to detect biomarkers are limited not by their ability to bind selectively to their target through e.g. an antibody or nucleic acid hybridisation, but instead by background signals inherent in their detection system reducing signal-to-noise ratios. One notable breakthrough in this regard has been shown using fluorescent nanodiamond labels with which external temporal modulation of the output signal can provide exceptional discrimination from background noise and a sub-attomolar detection limit (8 × 10^−19^ M) and single molecule identification of HIV RNA [58]. These properties, coupled with low toxicity and resistance to bleaching are driving multiple applications of nanodiamonds in quantum biosensing, including cancer biomarker analysis [59].

The large diversity both of biomarker analytes and the clinical applications of their analysis results in many diverse technologies being used in their study. Most of these technologies can be adapted and applied to the analysis of different analytes in healthy patients and those with diverse pathologies. Several technologies to analyse cancer biomarkers are well established: most commonly polymerase chain reaction (PCR)-based tests [60] to detect and quantify specific nucleic acids and forms of immunoassay, including enzyme-linked immunosorbent assay (ELISA) [61,62], to analyse proteins and metabolites. These techniques provide successful options for many applications, offer precise quantification and are versatile in their adaptation to new analytes and potential for multiplexing (e.g. Luminex assays). But they also have drawbacks. Their quantitation requires expensive hardware, they are unsuited to continuous monitoring, and they share drawbacks with many tests in their reliance on antibodies and enzymes with significant cost and limited storage stability. Additionally, standard immunoassay methods such as ELISA have sensitivity limits in the nanomolar to picomolar which is sufficient for many but not all clinical challenges. Techniques such as clinical mass spectrometry can generate rich datasets, for example, from plasma metabolites, but their use is limited by the need of costly equipment and highly trained personnel [63].

Partly for these reasons, the last two decades have seen increased research into the development for specific applications of cost-effective detection systems which are sensitive and more easily and directly quantifiable. In this regard, biosensors have been reported using technologies including electrochemical, electro-optical and Surface Enhanced Raman Spectroscopy (SERS), which also offers the capability of multi-analyte detection in small sample volumes [64]. Although optical and SERS-based detection methods enable multi-analyte analysis, their practical implementation is limited by factors such as complex instrumentation, the need for multiple excitation sources, tagging requirements, and long acquisition times. Alternatively, electrochemical detection technology shows promise for early cancer biomarker analysis as this offers lower limits of detection (into the femtomolar range), ease of use, and rapid read-out. Moreover, electrochemical detection technology allows the integration of such isolation or enrichment technologies onto the electrodes, which could potentially enable high sensitivity detection at the point-of-care.

### Electrochemical sensors for the analysing of circulating biomarkers

**Figure 2:**
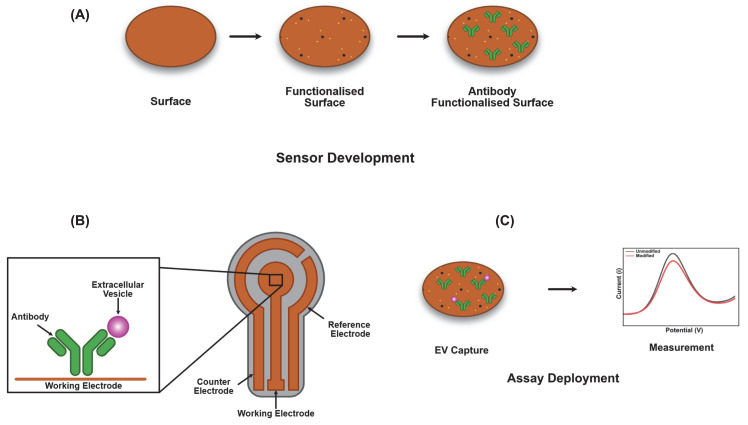
Electrochemical sensor development (**A**) The development of a model electrochemical sensor is shown, illustrating surface functionalisation and antibody attachment. An example of the spatial arrangement of a potential sensor is shown in (**B**) showing Working, Reference and Counter electrodes and a model assay signal profile in (**C**) showing the consequences on current versus voltage measurements of analyte binding to the antibody functionalised surface.

Electrochemical biosensors incorporating a biological recognition element along with a physicochemical transducer serve as rapid analytical detection tools for the precise detection of tumour associated nucleic acids, tumour protein markers, and EVs. Such sensors can be used as an effective tool for both screening of the disease as well as to monitor progression. A range of electrochemical biosensor strategies [65] were developed via adapting immunological principles. Typical biosensors consist of a biological recognition element (e.g. antibody, aptamer, and peptide) attached to a sensitive biosensor transducer element which usually consists of nanomaterials (Figure 2). When the biological recognition molecule encounters the appropriate analyte, the binding (e.g. antibody–antigen and DNA hybridisation) produces an multimolecular complex. The transducer converts this immune response process into detectable electrical signals and thus enabling both qualitative and quantitative protein detection. Here, label-free detection generates signals from one step immunoreaction, whereas labelled detection strategy or two-step immunoreaction (e.g. sandwich assays in which the antigen is sandwiched between the labelled-secondary antibody and a capture antibody immobilised onto the transducer), uses the signal generated from immunoreaction involving the labelled biological recognition molecule.

A wide range of approaches have been employed for the successful attachment of the biological recognition elements onto the desired transducer (biofunctionalization) [66,67]. The key aspects to be considered whilst choosing the biofunctionalization strategies are to preserve the biological activity of the biological recognition element, retain the inherent physicochemical properties of the transducer element, prolong the biosensors lifetime, and provide reusable and higher control over the orientation of the antibodies for higher detection capabilities. To achieve this, covalent bonding methods (e.g. EDC/NHS method [68,69] (N-ethyl-N′-(3-(dimethylamino)propyl) carbodiimide/*N*-hydroxysuccinimide) are widely used to immobilize antibodies onto the surface of transducers, whereas Au-thiol functionalisation strategies [70] are widely used in case of aptamers and peptide immobilisation. Additionally, a wide range of nanomaterials [71,72] (e.g. carbon or gold nanoparticles) have been employed as the transducer elements for electrochemical signal amplification, increase the loading density of biomolecular recognition elements and to enhance the charge transfer rates at the electrode/electrolyte interfaces.

### Protein biomarker analysis

In the past few decades, a wide range of proof-of-concept electrochemical sensor strategies have been reported demonstrating successful detection of protein biomarkers at or below relevant clinical concentration ranges. For instance, whilst the clinical gold standard tests for prostate cancer diagnosis demand the precise detection of prostate specific antigen (PSA) in plasma between 4 and 10 ng/ml [73,74], successful electrochemical successful of PSA has been shown with a detection limit down to 10 fg/ml (with a linear range between pg/ml and ng/ml) potentially extending the range of source biofluids [75–77]. Similarly, reports on high sensitivity detection of protein biomarkers including CA125 and CEA show ultra-low-level detections with wide linear range via employing diverse signal amplification strategies [78,79]. These include nanomaterial-assisted surface enhancement, enzymatic catalysis, nucleic acid-based amplification, redox-active tagging, and electrocatalytic processes—often combined with aptamer or antibody recognition elements. Additionally, the development of low-cost electrochemical sensors has been driven by advances in affordable nanomaterials, scalable fabrication methods such as screen-printing and inkjet printing, integration with disposable platforms (e.g. paper-based sensors) [80], and the use of portable, low-power electronics for signal readout. Thus, the current state-of-the-art electrochemical biosensors have outpaced the cancer diagnostics with most reports demonstrating the ultra-low-level detection of single cancer biomarker as a proof-of-concept. However, the current challenge relies in translating the technology for real world applications that needs rigorous analytical and clinical validation. Furthermore, due to the complexity of cancer, developing electrochemical sensors for measuring panels of biomarker is imperative.

To date, considerable efforts are devoted towards developing multiplex electrochemical sensors for cancer biomarker analysis, most of these strategies involve spatially separated electrodes, utilisation of multiple tags, and analysing multiple biomarkers [81–83]. Spatially separated electrodes provide the ability to selectively functionalise the individual electrode surfaces with the desired biological recognition elements, offering multiplex detection of various proteins. The current limitations in developing multiplex detection using a single electrode include (a) the ability to resolve electrochemical signatures generated by the probes, (b) steric effects, and (c) limitations in electrode surface functionalisation procedures. All these factors would affect the efficiency and performance of the electrochemical sensor that would limit their practical application.

### Extracellular vesicle analysis

Analysis of EV proteins has gained momentum in the past decade. Nanoparticle Tracking Analysis (NTA) is often used for exosome quantification to analyse their numbers and size in liquid media. High-throughput detection of EV surface proteins can be achieved using flow cytometry; however, most existing hardware limits its accuracy when detecting smaller vesicles (with a diameter <500 nm), therefore requiring specialist high sensitivity nano-flow cytometers and highly experienced users to achieve reproducible results [84]. Mass spectrometry and Western blot can analyse abundant EVs surface proteins but require a large sample volumes and complex procedures. Thus, these techniques, commonly used in research, have limited use in clinical settings [85,86]. Alternatively, fluorescence-based detection [87] and nano plasmonic sensors [88,89] have demonstrated high sensitivity detection of tumour specific EV surface proteins. Recently, Feng et al. demonstrated the real-time detection of tumour derived EVs from cancer patient plasma (detecting EV binding at 8.37 × 10^7^ EVs/ml) using concentric gradient nanoplasmonic sensors [90]. However, potential photo-bleaching and expensive instrumentation requirements can limit their practical application.

Electrochemical detection is a promising alternative to overcome these drawbacks and potentially enables high-throughput, frugal, and rapid analysis of EVs for diagnostic and therapeutic applications. Typically, researchers have adapted established bioassay strategies, demonstrated for the detection of soluble protein biomarkers, selectively to capture EVs localising these proteins onto an electrochemical sensor platform. Table 1 shows recent developments in electrochemical sensors for the detection of EVs tested with cultured cell line material. For instance, in 2018, Kilic et al*.* demonstrated a label-free electrochemical impedance biosensor, in which biotinylated anti-CD81 antibody was immobilized through streptavidin-biotin interaction onto a screen-printed gold electrode surface. Using such a simple platform and label-free strategy, this immunosensor quantified EVs over a wide linear range of 10^2^–10^9^ EVs/ml with a detection limit of 77 EVs/ml [91]. Huang et al*.* developed an electrochemical detection platform using anti-CD63 antibody as a capture probe and aptamers as detection probes and a combination of hemin/G-quadruplex DNAzyme-peroxidase reaction and complex rolling circle amplification to achieve signal amplification [92]. Liu et al*.* demonstrated an electrochemical aptasensor, where CD63 aptamer linked ferrocene was utilised to capture EVs specifically by CD63 protein on EVs, with a limit of detection of 4.82 × 10^5^ EVs/ml. Although this sensor showed a detection limit of ∼4.82 × 10^5^ EVs/ml, they had a narrow linear range of detection from 10^5^ to 10^6^ EVs/ml [93].

**Table 1:** Electrochemical sensors for EVs detection (validated with cell line-derived EVs)

Target EV surface protein	Biorecognition element	Cell lines (source for EVs)	Detection range (EVs/ml)	Limit of detection (EVs/ml)	Reference
CD63	Aptamer	MCF-7	5 × 10^5^ – 5 × 10^6^	4.82 × 10^5^	[93]
CD63	Aptamer	Hela Cells	5 × 10^5^ – 5 × 10^8^	2.29 × 10^5^	[94]
CD63	Aptamer	HepG cells	1 × 10^6^ – 1 × 10^9^	1 × 10^6^	[95]
CD81	Antibody	MCF-7	10^2^–10^9^	77 (EIS) 397 (DPV)	[91]
CD63	Aptamer	SGC7901	4.8 × 10^3^ – 4.8 × 10^6^	950	[92]
HepG2	Aptamer	HepG2	10^4^–10^13^	2.09 × 10^4^	[96]
EpCAM	Aptamer	HCT116	1.1 × 10^5^ – 1.1 × 10^8^	4.4 × 10^4^	[97]
EpCAM PSMA	Aptamer	VCaP U937	–	50	[98]
HER2	Antibody	BT-474 cell	4.7 × 10^8^ – 3 × 10^10^	4.7 × 10^8^	[18]

Most of the sensors reported so far have been presented in proof-of-concept demonstrations of selective and sensitive detection of EVs in spiked samples and using EVs secreted by cultured cells. A smaller number have been validated with clinical material (Table 2). Jeong et al. reported an integrated approach, iMEX (integrated magnetic–electrochemical exosome), using anti-CD63 labelled magnetic beads for exosome capture; whilst the capture exosomes were labelled with secondary antibodies against target protein markers (e.g. EpCAM or HER2) and HRP for electrochemical sensing. They demonstrated the successful detection of exosomes in plasma samples from ovarian cancer patients at a sensitivity of <10^5^ vesicles using very small sample volumes (10 μl) [99]. An et al*.* developed a magneto-mediated electrochemical aptasensor for the simultaneous analysis of multiple BrCa EV surface proteins (e.g. MUC1, HER2, EpCAM, and CEA proteins) and demonstrated that the levels of these proteins on BrCa patient-derived EVs were all higher than those on healthy individual-derived EVs [100].

**Table 2:** Electrochemical sensors for EVs detection validated with clinical material

EV surface protein target	Biorecognition element used	Cell line (EVs source used for validation)	Patient samples	Detection range (EVs/ml)	Limits of detection (EVs/ml)	Reference
CD63, EPCAM, CD24, CA125	Antibody	OV90, OVCAR3, OCVA420, and TIOSE6 cells	Ovarian cancer Plasma	10^4^–10^8^	3 × 10^4^	[99]
MUC1, HER2, EpCAM, and CEA	Aptamers	MCF-7, SK-BR-3, MDA-MB-231, and BT474	Breast cancer Serum	1.2 × 10^6^ – 1.2 × 10^10^	1.2 × 10^6^	[100]
CD63	Antibody	BT-474, SW-48	Colorectal adenocarcinoma Serum	1 × 10^5^ – 1 × 10^10^	1 × 10^5^	[101]
CD63	Aptamer	MCF-7	Breast cancer Serum	1.12 × 10^5^ – 1.12 × 10^11^	9.6 × 10^4^	[102]

## Future directions

Despite the promising performance in ultra-low-level detection of clinically significant cancer biomarkers with wide linear range and successes in other clinical areas, no electrochemical biosensors has yet been approved by the FDA or EMA for clinical use in cancer diagnosis. This is due to several challenges, including the need to demonstrate sensor specificity in complex and diverse clinical samples, and to facilitate data interpretation for clinical relevance in addition to product development and regulatory challenges [103,104]. Moving the best existing biosensor technologies into widespread clinical use will require interdisciplinary collaborations engaging scientists, engineers, clinicians, regulators, and the healthcare industry. Issues around product development such as high reproducibility and spatially separated electrode design development to enable multiplex detection can be addressed via adapting modern manufacturing approaches (e.g. nanofabrication techniques, laser induced graphene manufacturing, screen printing etc.) to achieve uniformity and minimise batch to batch variations. And the creation of standardised operating procedure for biofunctionalization approaches, and sensor testing should be prioritised to achieve regulate compliance. But additionally, other long term broad strategies should also contribute to delivering better-performing sensors which may achieve faster uptake.

### Strategies to improve electrochemical biosensors: synthetic biology and AI

Here, we have mostly discussed technologies which are tested for their performance in the analysis of single biomarkers (although they may then be applied in multiplex formats). Clinical practice is increasingly informed by multiomic data addressing multiple biomarkers and biomarker classes and future challenges include designing analysis platforms which are well-suited to the simultaneous affordable quantitation of multiple informative markers from different classes [105]. In parallel, it will be important to determine the clinical value provided by such multiomic analysis, e.g. whether genomes and transcriptomes associated with specific tumour-derived EV populations deliver better clinical outcomes than analysis of total circulating cfDNA and cfRNA [106].

Although many biosensors are developed using existing biological recognition motifs such as antibodies, enzymes or oligonucleotides, the design of novel recombinant analyte binding proteins and other applications of synthetic biology expand the opportunities offered by electrochemical sensors [107]. For example, by adding fluorescent proteins to both termini of two different bacterial amino-acid binding proteins immobilised on gold electrodes, Murugappan et al. were able to amplify the changes in protein conformation induced by specific binding to leucine or glycine and in turn the resultant output signal of each biosensor [108]. Similarly, ligand-dependent interactions between a purified transcription factor and its cognate electrode-immobilised DNA sequence can deliver effective sensors for these ligands. This is exemplified well by the work of Sankar et al. who show that increasing concentrations of the hormone progesterone in a sample will displace the hormone-binding transcription factor SRTF1 from surface-immobilised DNA, delivering changes in current detected in square wave voltammetry which are dependent on progesterone concentration [109].

Integration of microfluidics can assist controlled bio-interface generation which could reduce cross reactivity, biofouling and signal interference. Rusling's research group successfully demonstrated the measurement of 8-protein panel in serum using microfluidic integrated immunoarrays that can identify patients with prostate cancer and inform the need for a biopsy [110,111]. Additionally, microfluidic integration might also simplify sample enrichment/isolation process via enabling minimally invasive, low-cost, and rapid finger prick sample collection. Furthermore, recent progress in miniaturised hand-held potentiostat manufacturing enables linking with smart phones, offering user-friendly operation [112].

To address the intrinsic complexity of cancer diagnosis, a multi-disciplinary approach of integrating artificial intelligence (AI) and machine learning algorithms to electrochemical biosensor platforms [113] is critical to assist resolving the data analytics issues around real-time data interpretation, pattern recognition, predictive diagnostics and enable extraction of clinically relevant insights from complex datasets. Diverse research programmes aiming to identify specific disease-associated patterns within complex data are being accelerated greatly by artificial intelligence analysing datasets in applications such as multiparameter ctDNA sequence analysis [114]. Machine learning principals are also being applied to analyse electrochemical sensor current flow data and provide stronger identification of changes associated with target ligands or patient characteristics [115]. Recent relevant examples include the improved identification of bladder cancers through multi-sensor urine analysis [116] and more accurate analysis of cancer cell viability data in cytotoxic drug screening [117].

## Perspectives

- **Highlight importance of the field:** Current technologies support many important established and emerging clinical applications. New and better technologies have the potential to support cheaper biomarker measurements which are more detailed, richer and more informative and which can be made away from centralised testing facilities
- **Summary of the current thinking:** Cancer incidence is rising globally and many new treatments must be matched to specific patient groups. Therefore, better minimally invasive analysis of cancers will be required for the effective and affordable diagnosis, treatment and monitoring of cancer
- **Comment on future directions:** Biomarker detection sensitivity and selectivity can be improved through the application of synthetic biology and quantum technologies. Relatedly, improvements in machine learning and other forms of artificial intelligence allow the recognition of disease-associated patterns within complex, diverse datasets and are being used in many biomarker analysis technologies

## Open Access

Open access for this article is provided via our Subscribe to Open programme, made possible through the support of our subscribing institutions. Learn more here: https://portlandpress.com/pages/open_access_options_and_prices#conditional.

## Abbreviations

cfDNA: cell-free DNA
cfRNA: cell-free RNA
CTC: circulating tumour cell
ctDNA: circulating tumour DNA
EGFR: epidermal growth factor receptor
EpCAM: epithelial cell adhesion molecule
HER2: human epidermal growth factor receptor-2
PSA: prostate-specific antigen
NTA: Nanoparticle Tracking Analysis
TRPC5: transient receptor potential channel 5
